# Collisions of Two-Phase Liquid Droplets in a Heated Gas Medium

**DOI:** 10.3390/e23111476

**Published:** 2021-11-08

**Authors:** Pavel Tkachenko, Nikita Shlegel, Pavel Strizhak

**Affiliations:** Scientific and Educational Department of I.N. Butakova, Power Engineering School, National Research Tomsk Polytechnic University, 634050 Tomsk, Russia; ppt1@tpu.ru (P.T.); nik.shlegel.ask@gmail.com (N.S.)

**Keywords:** two-phase droplets, vapor bubbles, high-temperature gas medium, interaction, collision regimes, secondary fragments

## Abstract

The paper presents the experimental research findings for the integral characteristics of processes developing when two-phase liquid droplets collide in a heated gas medium. The experiments were conducted in a closed heat exchange chamber space filled with air. The gas medium was heated to 400–500 °C by an induction system. In the experiments, the size of initial droplets, their velocities and impact angles were varied in the ranges typical of industrial applications. The main varied parameter was the percentage of vapor (volume of bubbles) in the droplet (up to 90% of the liquid volume). The droplet collision regimes (coalescence, bounce, breakup, disruption), size and number of secondary fragments, as well as the relative volume fraction of vapor bubbles in them were recorded. Differences in the collision regimes and in the distribution of secondary fragments by size were identified. The areas of liquid surface before and after the initial droplet breakup were determined. Conditions were outlined in which vapor bubbles had a significant and, on the contrary, fairly weak effect on the interaction regimes of two-phase droplets.

## 1. Introduction

Two-phase gas-vapor-droplet flows are extensively used in power supply systems due to their high integral characteristics of heat transfer as compared with single-phase flows [1,2,3]. The processes developing from the collisions of droplets [4,5] containing gas or vapor bubbles are comparable to the micro-explosive destruction of liquid [6,7]. However, in that case, the second phase in a water droplet emerges as a result of fast boiling liquid passing through a pipeline [8] and continuing its movement in a high-temperature gas medium [9]. Gas bubbles can also end up in liquid droplets when they collide during spraying [10]. In a review of the application of different types of effervescent-swirl atomizers, employed air for spraying, Czernek et al. [11] described a group of contributing factors. On exiting the nozzle, the bubbles extend, leave the liquid without breaking it up, or burst to produce a group of secondary fragments. Similar processes occur when vapor bubbles leave a liquid droplet. Stierle et al. [12] studied the specific aspects of choosing a vapor-liquid interface when investigating phase transformations. The conditions typical of equilibrium modes and those far from them (high pressure and temperature characteristic of combustion chambers) were considered. The research [12] was conducted for two cases: a sharp interface and a diffuse interface. The main problems for these approaches in modeling were identified.

Air conditioning and refrigeration equipment, as well as the alternative energy industry use liquids whose properties differ significantly from those of water. Such liquids provide low-grade heat or make it possible to control the energy equipment temperature. To improve the performance of energy system components, it is necessary to carry out a more detailed investigation of the parameters of refrigerant evaporation and condensation. The evaporation of R134a refrigerant droplets was modelled by Zhou et al. [13]. The modeling results in [13] indicate that using a nozzle with a smaller diameter reduces the penetration depth of the aerosol flow, the size of a droplet, and its velocity. The research findings [14] reveal that the rate of evaporation and boiling heat transfer can be increased by the interaction of easily broken bubbles. Gas bubbles in moving droplets undergo transformation even on a short section of their way due to their movement and collisions, as shown by Xu et al. [15]. Li et al. [16] performed three-dimensional modeling of liquid film formation during liquid spray in industrial cooling systems. The calculations showed that at a high atomization rate, the composition of the aerosol cloud hardly changes when it moves away from the nozzle. In droplets of this size, heat exchange proceeds at a very high rate, and the local heating of liquid can lead to the formation of vapor bubbles. The problem of determining the surface temperature at the liquid–vapor interface should be brought to notice. Bedeaux and Kjelstrup [17] assumed that the temperature at the interface of two phases in a single-component liquid can be determined using the surface equation of state. The thermodynamic properties of multicomponent surfaces, presented in [17], make it possible to obtain the temperature at the liquid–vapor interface for single and multicomponent liquids. As pointed out by Bedeaux and Kjelstrup [17], this assumption requires experimental verification. Yet, it remains a challenge to conduct such experiments due to very fast processes and the small size of the region under study. Measuring the size of gas bubbles and their velocities in droplets, films, and layers of liquids using high-speed video cameras is complicated by light refraction effects emerging at the gas–liquid interface. The impact of this factor is usually minimized by adding fluorescent particles and using bandpass filter. Still, this approach does not provide a high accuracy of measurements [18]. Cerqueira et al. [18] developed a method of measuring the size and velocity of gas bubbles in liquid using fluorescent particles and a combination of particle image velocimetry (PIV) and laser-induced fluorescence (LIF) techniques. The accuracy of the results obtained is comparable to that of manual measurement. The research [18] was followed up by developing a neural network algorithm to identify bubbles and determine their shape [19]. The network trained by the images of bubble movement in water showed good results when processing bubble movement images in glycerol too. The findings of Cerqueira et al. [18,19] make it possible to dramatically accelerate the video frame processing and obtain experimental results faster and with high accuracy.

In addition to heat exchange equipment, there are other industries where bubble effects are present. Qin et al. [20] explored the effect of gas bubbles in mineral oil on its breakdown in energy equipment. The experimental research into the collisions of gas bubbles is fraught with the difficulty of obtaining accurate data, as described above, due to light refraction at the gas–liquid interface. Zhang et al. [21] used modeling to study the coalescence of two identical sphere-shaped gas bubbles. Four coalescence regimes conditioned by the movement of liquid around bubbles were identified. The research findings made it possible to establish that a decrease in viscosity shortened coalescence, as liquid layers took less time to mix in the droplet.

When fuel droplets move in a gas medium, they do not only evaporate but also ignite at a high temperature. Ashna and Rahimian [22] developed a model to investigate the head-on collision of two evaporating or burning fuel droplets in a coalescence regime. The modeling results [22] were compared with the data of the experimental study of an n-decane droplet combustion conducted by Dietrich et al. [23]. From the results obtained using the model, Ashna and Rahimian [22] drew a conclusion that the coalescence of evaporating droplets required lower Weber numbers than under the normal conditions. In the coalescence of burning droplets, slight acceleration of their velocity was detected just before the collision, which is caused [22] by a decrease in the gas density due to flame combustion. A significant limitation of the model [22] is that it is implemented only using two-dimensional setups.

The purpose of this study is to use the experimental results to establish the impact of the proportion of vapor in two-phase liquids on the critical conditions of regimes and on the characteristics of droplet collisions.

## 2. Materials and Methods

**Figure 1:**
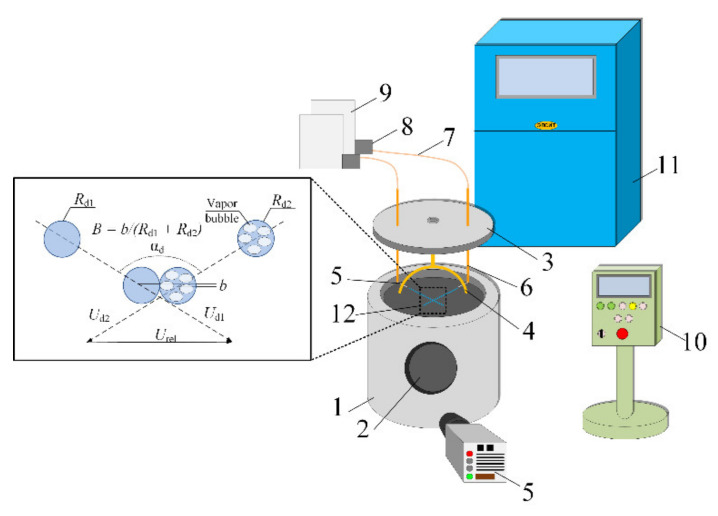
Schemes of setup and droplet collision parameter recording: 1—metal cylinder chamber; 2—observation window; 3—lid; 4—ring-shaped holder; 5—nozzles; 6—copper tubes; 7—silicone tubes; 8—pumps; 9—containers with liquid; 10—induction heater control panel; 11—liquid cooling system; 12—recording area.

In the experiments, a setup schematically shown in Figure 1 was used. The main element of the setup was a 40 kVA induction heater. In the inductor, a metal cylinder chamber was installed with two observation windows with a diameter of 120 mm, mounted exactly opposite each other. Quartz glass was set in the observation windows to withstand high temperatures and not crack when water droplets hit them. In the bottom part of the cylinder chamber, a liquid drain valve was mounted to eliminate high vapor volume fraction.

The top of the cylinder chamber was covered with a lid into which hose nipples were fitted to connect to copper tubes for liquid supply. A ring-shaped holder for nozzles was attached to the lid from the inside. Such an approach made it possible to record the nozzle position and prevent a relative shift of droplet flows due to the deformation of metal when it was heated to high temperatures. Two chromel–alumel thermocouples (with a measurement range of −50–1200 °C, systematic error of ±1.5 °C, and junction diameter of 0.3 mm) were installed in the metal cylinder to measure the temperature of its walls and the gas medium inside it. This setup allowed heating the gas medium in the cylinder chamber to 950 °C. To prevent the superheating of the induction heater, a closed-loop liquid cooling system was employed. Antifreeze circulated in the loop.

Droplet collisions were recorded by a high-speed video camera. The experiments were recorded with 768 × 576 pix resolution and 5000 fps film rate. Additional illumination of the recording area was provided by a 150 W LED spotlight.

Droplet flows were generated by two nozzles 0.84 mm in the internal diameter. The nozzles were arranged in the same plane at an angle of 120° to each other. The impact angle (α_d_) was varied from 30 to 90° and droplet radii (*R*_d1_, *R*_d2_) ranged from 0.1 to 1.3 mm. Droplet velocities (*U*_d1_, *U*_d2_) were varied from 1.2 to 4 m/s. The parameters of droplet movement and collisions were determined using the scheme presented in Figure 1. The relative velocity of the liquid droplet interaction was given by relative velocity.

*U*_rel_ = (*U*_d1_ + *U*_d2_ − 2·*U*_d1_·*U*_d2_·cos(α_d_))^1/2^

The Weber number (We_1_) ranged from 0 to 600 and was calculated accounting for the relative velocity of droplets. The Weber number of droplets with vapor bubbles (We_2_) was calculated after the density of such droplets was determined using the formula:

ρ_2_ = ρ_water_ (1 − γ) + ρ_vapor_·γ

The linear parameter of droplet interaction ranged from 0 to 1.

*B* = *b*/(*R*_d1_ + *R*_d2_)

The air temperature in the cylinder (*T*_g_) was maintained in the range of 400–460 °C. It dropped by 10–30 °C after water was sprayed. Therefore, the air temperature before the experiment is used further in the text.

The experiments were conducted with distilled water supplied from one container and with water containing some surfactant (Tween-80 with a volume concentration of no more than 0.5%). This reduced the surface tension of the composition and provided its faster evaporation in the heated gas medium as compared with water.

Four interaction regimes (bounce, coalescence, separation and disruption) were analyzed in the video frame processing [24,25,26]. In bounce, the elastic collision of droplets does not break their surface layer. No new fragments are produced, and the droplets continue moving independently. Coalescence is when two droplets merge to continue moving as a single droplet. Separation was recorded, when droplets merged and split up into two single droplets without producing new fragments. Disruption occurs with the intense atomization of initial droplets into numerous child droplets (*N*_ti_ > 2) after the collision. Two kinds of disruption were recorded: with a liquid disc and with a bridge between the droplets. These are conditioned by the impact centricity. Displaced impact (*B* ≥ 0.6) led to the disruption with a liquid bridge between the droplets.

The integral characteristics of newly formed secondary fragments were investigated. For that, all the recorded secondary (child) droplets were counted (*N*_ti_) and their radii (*r*_d_) were measured. The volumes of droplets before the collision and child droplets formed after the interaction were calculated to control the conservation of mass of liquid.

*V*_0_ = 4·π·(*R*_d1_^3^ + *R*_d2_^3^)/3

*V*_1_ = 4·π·Σ*r*_di_^3^/3

We took into account that some liquid could evaporate while the droplet was moving in a heated gas medium. To calculate the liquid volume in a two-phase droplet, we subtracted the volume of vapor areas from the total droplet volume:

*V*_v_ = 4·π·Σ*r*_vi_^3^/3

*V*_d_ = 4·π·*R*_d_^3^/3

After that, we calculated the volume of liquid and volume fraction of vapor in the droplet:

*V*_liq_ = *V*_d_ − *V*_v_

γ = *V*_v_/*V*_d_·100%

Then, the size of the newly formed fragments was proportionally increased so that *V*_0_ equalled *V*_1_. This allowed us to take into account the size measurement error due to the non-sphericity of secondary fragments and deformation of the liquid surface. Finally, we calculated the ratio (*S*_1_/*S*_0_), i.e., the free surface area of newly formed child droplets to that of pre-collision droplets:

*S*_1_ = 4·π·Σ*r*_di_^2^

*S*_0_ = 4·π·(*R*_d1_^2^ + *R*_d2_^2^)

## 3. Results and Discussion

**Figure 2:**
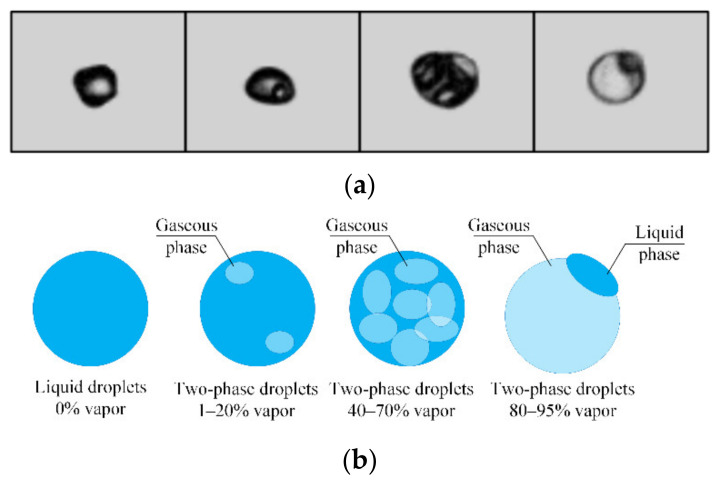
Video frames (**a**) and schematic image (**b**) of droplets with different relative volume fraction of vapor bubbles in them.

The video frame processing revealed four types of droplets with different relative volume fraction (proportion) of vapor bubbles in them. The schematic image of droplets and their appearance in video frames are presented in Figure 2. The first type is single-phase liquid droplets (without bubbles) (Video S(**A**): Water droplet (left and right) with 0% of vapor.). The second type of droplets with a low relative volume fraction of vapor (which can be calculated by comparing the total bubble volume with the droplet volume) is typical of cases when water is superheated when moving through a copper tube and nozzle (Video S(**B**): Water droplet (left) and two-phase droplet with 30% of vapor (right)). The boundary layer of liquid evaporated after contacting the heated tubes. Due to the turbulent flow of water, the emerging vapor ended up in the droplets as a group of bubbles. In some cases, up to three vapor bubbles in the droplet were recorded. The third and fourth types of droplets are typical of the aqueous solution of the surfactant Tween-80 (Video S(**C**): Water droplet (left) and two-phase droplet with 60% of vapor (right) and Video S(**D**): Water droplet (left) and two-phase droplet with 93% of vapor (right)). Lower surface tension of the composition caused it to evaporate faster. The formation of droplets with a relative vapor proportion of 50% and above goes to show this fact. In exceptional cases, the fragments of water with a vapor volume fraction of more than 50% were recorded. In most cases, two-phase droplets with a vapor volume fraction of more than 50% were recorded after the addition of a surfactant to water. The established persistent aspects were used to plot curves for four groups of droplets with different vapor volume fractions (Figure 2b): (1) two water droplets without vapor bubbles; (2) droplets with a relative vapor volume fraction of 1–20%; (3) droplets with a vapor volume fraction of 40–70%; (4) droplets with a vapor volume fraction of over 80% (they were usually one large vapor bubble with a liquid film of different thickness).

**Figure 3:**
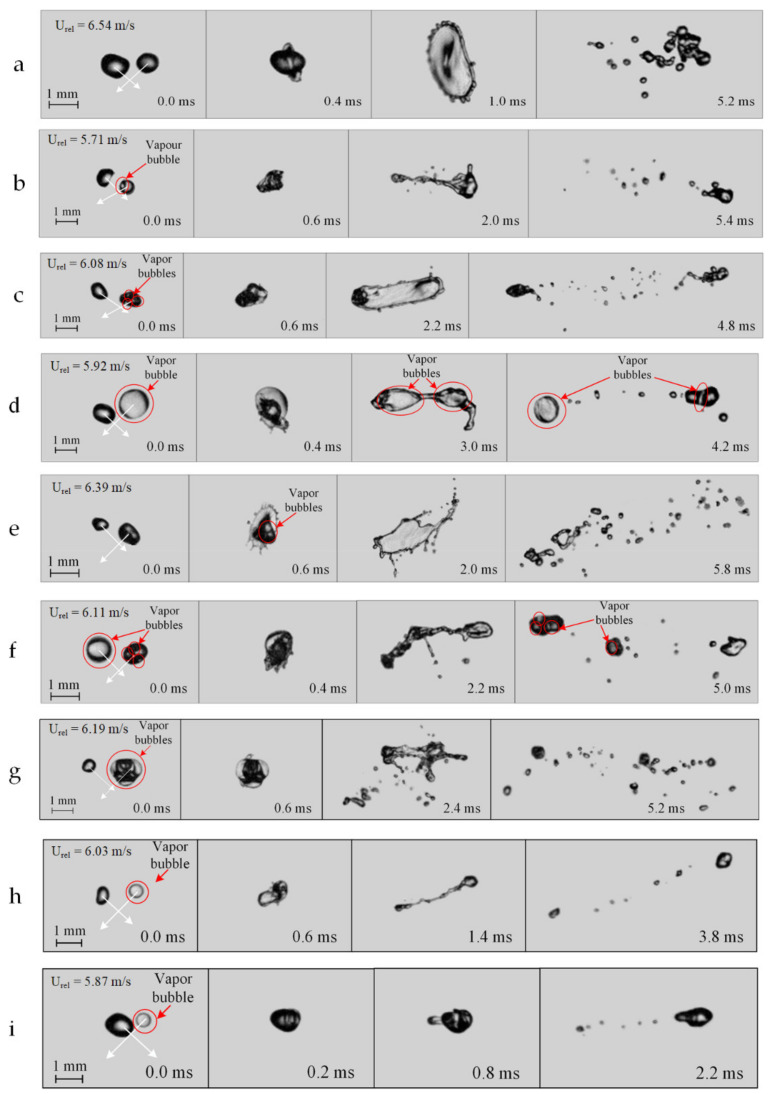
Video frames of colliding liquid droplets with different proportion of vapor inside them: (**a**)—two liquid droplets without bubbles; (**b**)—a liquid droplet without vapor (left) and a droplet with 10–20% bubbles (right); (**c**)—a liquid droplet without vapor (left) and a droplet with about 60% bubbles (right); (**d**)—a liquid droplet without vapor (left) and a droplet with about 90% vapor (right); (**e**)—a liquid droplet without vapor (left) and a droplet with about 10% vapor (right); (**f**)—a droplet with about 90% vapor (left) and a droplet with about 20% vapor (right); (**g**)—a liquid droplet without vapor (left) and a droplet with about 70% bubbles (right) at a size ratio Δ ≈ 0.5; (**h**)—a liquid droplet without vapor (left) and a droplet with about 90% vapor (right) at a size ratio Δ ≈ 1; (**i**)—a liquid droplet without vapor (left) and a droplet with about 90% vapor (right) at a size ratio Δ ≈ 2.

Typical video frames of the collisions of droplets sized about 1 mm with different proportions of vapor in them are presented in Figure 3. The most frequent outcomes in the conducted experiments are presented. The main interaction regime was disruption of two-phase droplets. The proportion of vapor bubbles in the droplets had a significant effect on their disruption characteristics. When droplets with a low proportion (less than 10%) of vapor collided (Figure 3a,b), their disruption resembled that of two colliding droplets without vapor bubbles. Their subsequent movement as an aerosol cloud and enlargement due to coalescence produced a cloud of 7–20 secondary fragments sized from 0.07 to 0.5 mm. A rise in the number and volume of vapor bubbles (Figure 3c) increased the number of secondary fragments to 30–40. Yet, during markedly off-center impact (*B >* 0.5), a pair of large (up to 0.8 mm) vapor droplets was formed in addition to fragments sized 0.07–0.2 mm.

**Figure 4:**
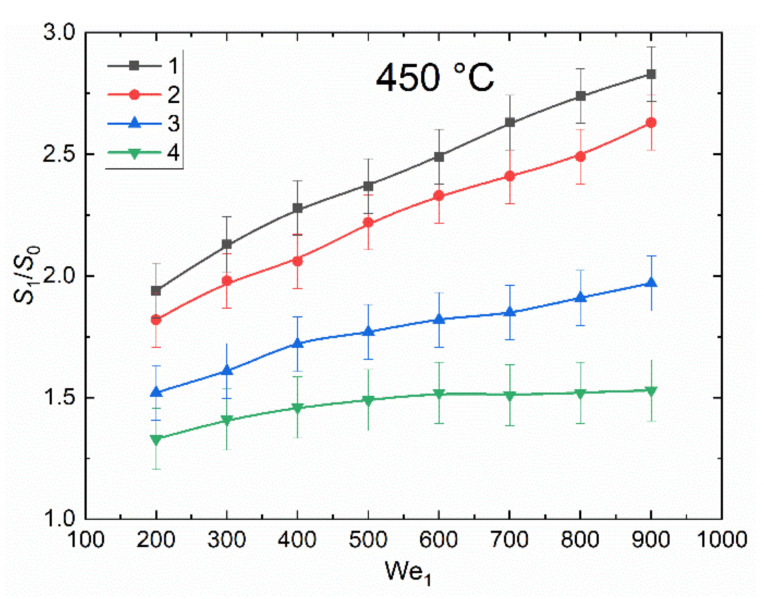
The ratio of the free surface area of liquid before and after the interaction of droplets with different proportion of vapor bubbles vs. the Weber number (We_1_), calculated for the water droplet: 1—two droplets without vapor; 2—a droplet without bubbles and a droplet with about 10% vapor; 3—a droplet without bubbles and a droplet with about 60% vapor; 4—a droplet without bubbles and a droplet with about 90% vapor.

**Figure 5:**
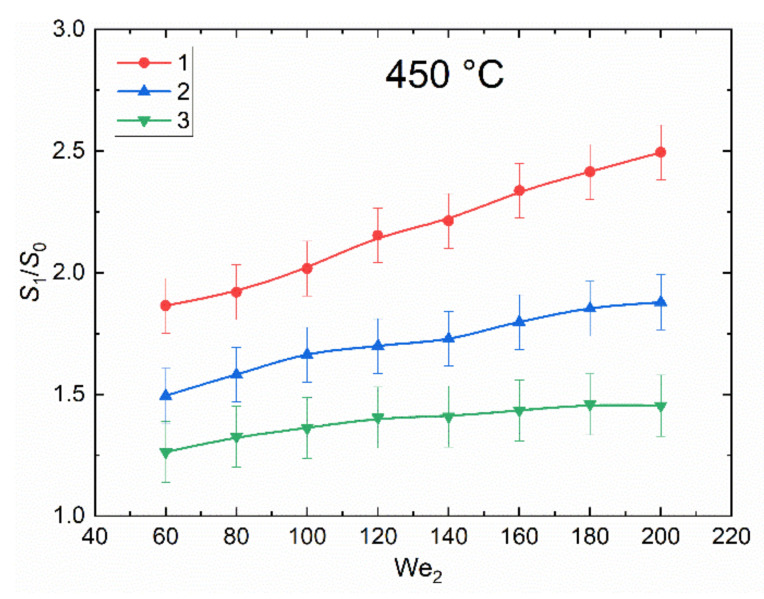
The ratio of the free surface area of liquid before and after the interaction of droplets with different proportion of bubbles vs. the Weber number (We_2_), calculated for the two-phase droplet: 1—a droplet without bubbles and a droplet with about 10% vapor; 2—a droplet without bubbles and a droplet with about 60% vapor; 3—a droplet without bubbles and a droplet with about 90% vapor.

Figure 4 presents the curves of the ratios of the free surface area of liquid before and after the interaction of droplets with different proportion of bubbles for the Weber number range from 200 to 900. When determining the ratio of free surface areas of liquid before and after collision, the Weber number has been chosen as the key dimensionless parameter, since it shows the ratio of the inertia forces of the liquid to the surface tension. The criterion allows us to evaluate the dynamics of changes in the values of *S*_1_/*S*_2_ with changes in the properties of the liquid, the size and velocity of the droplet. The curves were plotted for the collisions of four droplet types under study, described above and schematically shown in Figure 2. For the droplets with 0 to 20% vapor, the growth of the free surface area before and after the interaction was minimal. This is because the energy in the liquid when two droplets collide is evenly distributed in all directions. A low proportion of vapor in droplets has a negligible effect on energy propagation. When the vapor volume fraction in droplets is high (over 40%), the vapor cavities in them absorb some energy in the collision zone due to compression and extension. This leaves less energy for the separation of liquid layers in the droplet. The ratio *S*_1_/*S*_0_ decreases with a rise in the volume fraction of vapor bubbles in droplets. The curves presented in Figure 5 are similar to those in Figure 4, though in this case the Weber number (We_2_) was calculated for the droplet with vapor bubbles. Since the density of vapor is significantly lower than that of water, the Weber numbers here were much lower than in the case of the water droplet without vapor bubbles.

The values error bars in Figure 4 and Figure 5 have been determined by fluctuations in the size of droplets and the vapor volume fraction in them, since at this stage it is quite difficult to generate absolutely identical two-phase droplets. Moreover, when droplets collide, the impact may first occur on the side of a two-phase droplet containing a larger layer of water, and not on a thin liquid film. In this case, at the initial stage of interaction, the droplets mainly behave as in the case of a collision of single droplets. A certain instability is caused by a change in the shape of droplets when they move in a high-temperature gas environment. Fluid fluctuations in them affected collisions. Another reason for fluctuations in the values of *S*_1_/*S*_2_ is the value of the scale factor, which introduces a constant error in determining the dimensions.

**Figure 6:**
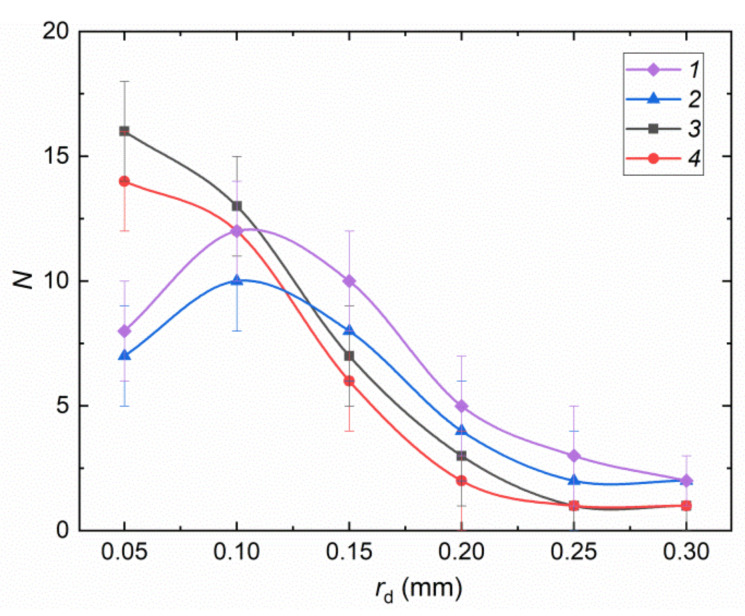
Distribution of secondary liquid fragments after the disruption of two colliding droplets with different proportion of bubbles: 1—two droplets without bubbles; 2—a droplet without bubbles and a droplet with about 10% vapor; 3—a droplet without bubbles and a droplet with about 60% vapor; 4—a droplet without bubbles and a droplet with about 90% vapor.

The distribution of liquid child droplets is presented in Figure 6. In the vast majority of the conducted experiments, when droplets with *B* > 0.2 collided, they broke up to produce a chain of different-sized secondary fragments. In this case, the child droplets of all the liquids under study were represented by two relatively large fragments (*r*_d_ > 0.3 mm) and several small ones (*N*_ti_ = 2–9). The distribution extremum was attained near the value *r*_d_ = 0.1 mm. The number of droplets with *r*_d_ = 0.15–0.3 mm ranged from 15 to 30. The secondary fragment distributions obtained suggest that the relative fraction of vapor in droplets may be used as the initial parameter for varying child droplet characteristics together with *B* and We.

When analyzing the experimental video frames, we outlined the conditions in which vapor bubbles had a significant and, on the contrary, fairly weak effect on the interaction regimes of two-phase droplets. It was established that vapor bubbles had the most significant effect on the interaction regimes when the size of the droplet with bubbles was much smaller than that of a single-phase droplet. In this case, different regimes may occur, depending on the vapor bubble volume fraction in the first droplet. In particular, when the vapor bubble volume fraction was maximum and the single-phase droplet size was minimal, the latter penetrated the near-surface layer of the two-phase droplet and ended up in its volume (inside the bubble). This interaction regime can be regarded as coalescence. If the velocity of the small single-phase droplet was severalfold higher than that of the two-phase droplet, separation was recorded (the smaller droplet penetrated the vapor bubble and went all the way through it). It is important to note that the vapor bubble was not always destroyed by such an interaction. If the bubble was severalfold larger than the liquid droplet that broke through it, the holes made in its envelope quickly decreased and the envelope was restored. The bubble reduced in size, though, because some vapor escaped from it. If the size of the bubble was 1.5–2.5 times as large as the approaching single-phase droplet of liquid, it was destroyed as a result of the collision. In the experiments with comparable single-phase and two-phase droplets, disruption and bounce were recorded depending on the relative velocities and coaxial alignment of the collision. Thus, bounce was recorded at droplet velocities corresponding to We < 5. At We > 50, a group of child droplets was formed, which is typical of disruption. The analysis of collisions of two-phase droplets with each other revealed that the interaction regimes largely depended on the relative velocity and coaxial alignment of the collision. The lower the value of We and the higher the value of *B*, the more frequently two-phase droplets bounced, i.e., vapor bubbles did not collapse. A decrease in *B* led to separation or disruption. The higher We was, the more frequent was disruption. Coalescence was recorded only in those experiments where at least one of the two droplets was single-phase. This happens because the merging of two-phase droplets is slowed down by the pressure forces inside the bubbles. These forces repel the bubbles and the near-surface layers of droplets from each other. More importantly, at virtually any parameters, the interaction of a large single-phase droplet with a small two-phase one proceeds with the disruption of the latter or collapse of bubbles and merging of the two-phase droplet envelope with the single-phase droplet. Thus, the interaction in the video frames looked like a vapor or air bubble interacting with a solid surface. At a low relative velocity, a two-phase droplet as a bubble with a vapor envelope contacts the solid surface, the bubble collapses, and the liquid spreads over the surface. If the interaction rate was increased, the two-phase droplet broke up from the collision to produce a number of small fragments. When generalizing the research findings, we produced a map of droplet collision regimes. The error bars in Figure 6 are a consequence of the reasons described earlier in the text for Figure 4 and Figure 5. The number of secondary fragments formed is also affected by the part of the drop into which the water droplet hits, and the shape of both drops.

Some specific aspects of collisions of droplets with bubbles and without them are also noteworthy (Figure 3g–i). When a liquid droplet is significantly smaller than a two-phase droplet, the former is highly likely to stick to the surface of the latter because the smaller droplet slows down on the surface of the larger droplet due to its elastic compression. Yet at a relatively high velocity (over 3–4 m/s), the smaller droplet may penetrate the vapor bubble or destroy it (disruption). When the two-phase droplet is significantly smaller than the liquid droplet, it is usually destroyed after the collision. When two-phase and homogeneous droplets have comparable sizes, the collision regime depends largely on the relative velocity. The higher this velocity, the more often disruption occurs. It is also clear from Figure 3g–i that with the growing ratio of the size of the liquid droplet without vapor to that of the droplet with vapor, the number of secondary fragments resulting from the disruption of the initial droplets decreased. These secondary fragments were highly heterogeneous in terms of their component composition (i.e., the video frames clearly show the content of water and bubbles) (Figure 3c,d,f).

The analysis of the video frames of the conducted experiments reveals that vapor bubbles have a significant impact on the shape of droplets before and after their interaction. In particular, the higher the volume fraction of vapor bubbles in the droplet, the more significant the transformation of its surface. The shape changed from spherical to ellipsoidal and geometrically complex ones. Consequently, the interaction of droplets with such a complex shape was characterized by the formation of a large number of secondary fragments. The latter became spherical faster if there were vapor bubbles in the composition. That happened because the bubbles in the composition of a small-sized droplet displaced liquid into the envelope layers. As a result, the droplet became spherical. The fewer bubbles there were in the composition of initial droplets before the collision, the more spherical the shape they assumed. The patterns established for the effect of the shape of two-phase droplets on interaction regimes and outcomes are generally consistent with the experimental results in [27]. Moreover, vapor cavities in droplets contributed to more significant deformation before their destruction due to partial vapor compression as compared with the collisions of liquid droplets without vapor. In the latter case, the deformation of droplets occurred only when they changed their shape due to the incompressibility of liquid.

The analysis of the video frames revealed interesting combined effects emerging from the successive collision of one droplet with several neighboring ones. The most interesting patterns were established in the successive interaction of a two-phase droplet with several single-phase ones, and, on the contrary, of a single-phase droplet with several two-phase ones. The video frames showed that a two-phase droplet underwent more significant surface transformation after it interacted with a single-phase droplet. Each subsequent interaction led to more significant surface transformation. At the same time, each collision displaced some part of vapor from the droplet. As a result, after 3–5 successive collisions, vapor bubbles were completely displaced from the droplet, which means that the interaction of droplets with each other may be used as a degassing method. In the experiments with successive interactions of a single-phase droplet with a group of two-phase ones, on the contrary, vapor bubble growth was recorded in the initial droplet. This indicates that the droplet is filled with small bubbles. Yet, the concentration of vapors in such droplets did not increase significantly after 2–3 collisions as they were very unstable. Therefore, by crossing jets with different content of vapor bubbles, it is possible to form aerosol clouds with droplets containing a predictable and controllable number of bubbles.

The analysis of thermal conditions, performed for the interaction of two-phase and single-phase liquid droplets, revealed that the evaporation of liquid plays an important role in the formation of secondary fragments and approach of the initial droplets. In particular, the higher the temperature of the external gas medium, the more significantly droplets slow down their movement before collision. A buffer vapor layer between droplets, analyzed in detail in [28], has a major part to play here. In contrast, with single-phase droplets studied before, the formation of a buffer vapor layer between two-phase droplets in the experiments proceeded more slowly. There are several reasons for that. First, two-phase droplets have a complex surface configuration. Their interaction proceeded with the formation of a complex droplet interface. Second, droplets contained bubbles distributed in the volume. As a result, the interaction was accompanied by the redistribution of bubbles in the near-surface layer. Third, due to significant deviation of the surface from the spherical shape, the aerodynamic resistance was higher for two-phase droplets than it was for single-phase ones. Significantly, the collapse of bubbles and evaporation of liquid from the free droplet surface considerably decreased the size of secondary fragments. Therefore, high-temperature blending of two-phase droplet flows may help to form highly reactive gas-vapor-droplet mixtures.

The integral characteristics of two-phase droplet atomization provide new insights into the collision of liquid droplets in a high-temperature gas medium, researched in [24,25]. Heating the gas medium to 400–500 °C made it possible to achieve critical (meta-stable, in particular) conditions of droplets. A high heat flux directed inside the droplet contributed to faster boiling of the liquid and the formation of vapor bubbles. In some cases, the heat flux value was so high that it led to partial micro-explosive breakup of the droplet as the vapor bubble escaped from it. Thus, we recorded previously unknown specific aspects of droplet movement and disruption in a high-temperature gas medium. As noted in the previous papers [24,25], droplets encountered resistance of the vapor region around them immediately before the collision. The high velocity of droplets contributed to partial entrainment and densification of vapors in the area in front of the flying droplet. This, in turn, affected droplet collision outcomes too. The presence of a vapor layer, though, built up additional resistance before the droplet collision.

## 4. Conclusions

As a result of studying the collisions of two-phase liquid droplets in a heated gas medium, the effect of vapor bubbles on their interaction regimes and outcomes was established. Two-phase droplets with different volume fractions of vapor bubbles in them (from 10% to 95%) were used. The collision regimes of two-phase droplets were defined. Specific aspects of the disruption of four two-phase droplet types were described when they collide with water droplets without bubbles. Droplets with a high vapor volume fraction (more than 60%) when colliding with water droplets were crushed into many secondary fragments, similar to the micro-explosion regime, which has been of great interest recently. With the help of integral characteristics, qualitative values of increasing the free surface area of droplets after crushing, which are important for improving the efficiency of industrial installations, are obtained. It was also found that vapor bubbles have the most significant effect on the interaction modes in the case when the size of a two-phase droplet is much larger than a single-phase liquid droplet. Maximum ratios of the free surface area of liquid fragments before and after droplet collisions were determined. Maximum values of *S*_1_/*S*_0_ were obtained for the collisions of two water droplets (up to 2.8). Minimum values were determined for water droplets colliding with droplets containing 80–90% vapor (a large vapor bubble) (up to 1.4). Secondary liquid fragments were distributed by size. These distributions can be used when choosing the parameters of secondary droplet atomization systems. Due to the fact that studies in this direction are difficult to implement and have some difficulties in reliably assessing the effect of the vapor volume fraction in single two-phase droplets when they collide, the results presented in this paper are not yet used in practical applications but have good prospects for further development of research in this direction.

## Data Availability

Data is contained within the article.

## Supplementary Materials

The following are available online at https://www.mdpi.com/article/10.3390/e23111476/s1, Video S(**A**): Water droplet (left and right) with 0% of vapor; Video S(**B**): Water droplet (left) and two-phase droplet with 30% of vapor (right); Video S(**C**): Water droplet (left) and two-phase droplet with 60% of vapor (right); Video S(**D**): Water droplet (left) and two-phase droplet with 93% of vapor (right).

## Nomenclature

Greek symbols	
α_d_	impact angle, °;
β	dimensionless angular interaction parameter;
γ	percentage of vapor in the droplet, %;
Δ	ratio of the radius of the droplet without vapor to that of the droplet with vapor;
µ	dynamic viscosity, Pa∙s;
ρ_Water_	water density, kg/m^3^;
ρ_v_	vapor density, kg/m^3^;
σ	surface tension of liquid, N/m.
Latin letters	
*b*	linear approach parameter, mm;
*B*	dimensionless linear parameter of interaction;
*N*	number of child droplets;
*r*_d_	radius of child droplets, mm;
*r*_v_	radius of the vapor region (as a group of bubbles) in the droplet, mm;
*R*_d1_, *R*_d2_	radii of interacting droplets, mm;
*S*_0_, *S*_1_	total surface area of liquid before and after interaction, m^2^;
*S*_1_/*S*_0_	ratio of free surface areas of liquid before and after collision;
*U*_d1_, *U*_d2_	velocities of interacting droplets, m/s;
*U*_rel_	relative droplet velocity, m/s;
*V*_0_	droplet volume before interaction, m^3^;
*V*_1_	droplet volume after interaction, m^3^;
*V*_liq_	liquid volume in a two-phase droplet, m^3^;
*V*_v_	vapor volume in a two-phase droplet, m^3^;
*V*_d1_	volume of a two-phase droplet, m^3^;
We_1_	Weber number for a water droplet;
We_2_	Weber number for a droplet with vapor bubbles.
Abbreviations	
BO	bounce;
CO	coalescence;
DI	disruption;
SE	separation.
